# The complete mitochondrial genome of *Axis porcinus* (Mammalia: Cervidae) from Victoria, Australia, using MiSeq sequencing

**DOI:** 10.1080/23802359.2017.1357451

**Published:** 2017-07-26

**Authors:** Erin Hill, Adrian M. T. Linacre, Simon Toop, Nicholas P. Murphy, Jan M. Strugnell

**Affiliations:** a Department of Ecology, Environment and Evolution, La Trobe University, Melbourne, Australia;; b School of Biological Sciences, Flinders University, Adelaide, Australia;; c Game Management Authority, Melbourne, Australia;; d Centre for Sustainable Tropical Fisheries and Aquaculture, Marine Biology and Aquaculture, James Cook University, Townsville, Australia

**Keywords:** Complete mitochondrial genome, hog deer, *Axis porcinus*, MiSeq sequencing

## Abstract

The mitochondrial genome of the hog deer (*Axis porcinus*) was sequenced using an Illumina MiSeq. The assembled genome consists of 16,351 bp, and shared a 99.8% similarity to the published chital deer (*Axis axis*) genome, suggesting that they belong to the same species. Further research is ongoing to understand why these mitochondrial genomes are highly similar.

The hog deer (*Axis porcinus*) was introduced into Australia in the nineteenth century by Acclimatisation Societies, primarily for hunting purposes (Mayze and Moore 1990). It is now endangered throughout its native range of Pakistan, India, Nepal, and Bangladesh, however, a stable population exists in the Gippsland region of Victoria, Australia (Scroggie et al. 2012). There is currently limited genetic knowledge of the population diversity, structure, and origin of the Australian *A. porcinus* population.

Presented here is the mitochondrial genome of *A. porcinus* from Victoria, Australia, sequenced via next generation sequencing methods. Liver samples were collected from four hog deer on Sunday Island, Victoria, Australia during the 2015 hog deer hunting season (−38.711683° 146.623333°). Genomic DNA was extracted using a DNeasy Blood and Tissue Kit (Qiagen, Hilden, Germany) following the manufacturer’s instructions. NebNext Ultra Library Prep Kit (BioLabs, Ipswich, MA) was used to create pair-end libraries, with size selection of 500–600 bp. The MiSeq Reagent Kit v3 (2x300) (Illumina, San Diego, CA) was used to prepare samples for sequencing on an Illumina MiSeq. The reads obtained from the MiSeq run were then processed using the program *Kraken v0.10.5* (Wood and Salzberg 2014) to remove non-target DNA sequences, resulting in an average of 5 million reads per sample. *CLC Genomics Workbench 7* (CLC bio, Inc., Aarhus, Denmark) was used for *de novo* assembly, with a minimum contig size of 1000. Protein coding regions and ribosomal sequences were determined using *MITOS* (Bernt et al. 2013).

Mitochondrial genomes of 12 closely related deer species were downloaded from GenBank, in addition to the *A. porcinus* mitochondrial genome from Australia. Phylogenetic analyses were performed using the maximum-likelihood method. All genomes were aligned using *Geneious 9.0.5* (Kearse et al. 2012). The best-fit model GTR + G was determined using *JModelTest 2.1.7* (Darriba et al. 2012), under the Akaike Information Criterion (Akaike 1974). A phylogenetic tree was created using the program RAxML (Stamatakis 2014), with 1000 bootstrap replicates. The *Muntiacus muntjak* (AY225986) mitochondrial genome was used as an outgroup.

The length of the *A. porcinus* genome was 16,351 bp with the base composition of A (33.4%), T (29.3%), C (24%), and G (13.3%) (GenBank Accession no. MF435989). The genome includes 13 protein coding genes, two rRNA genes, and 22 tRNA genes.

The phylogenetic tree shows that the Victorian *A. porcinus* and *A. axis* (JN632599) form their own clade, separate from the other *A. porcinus* genome (JN632600) (Figure 1). Bootstrap values show 100% support for both *Axis* clades.

**Figure 1.**
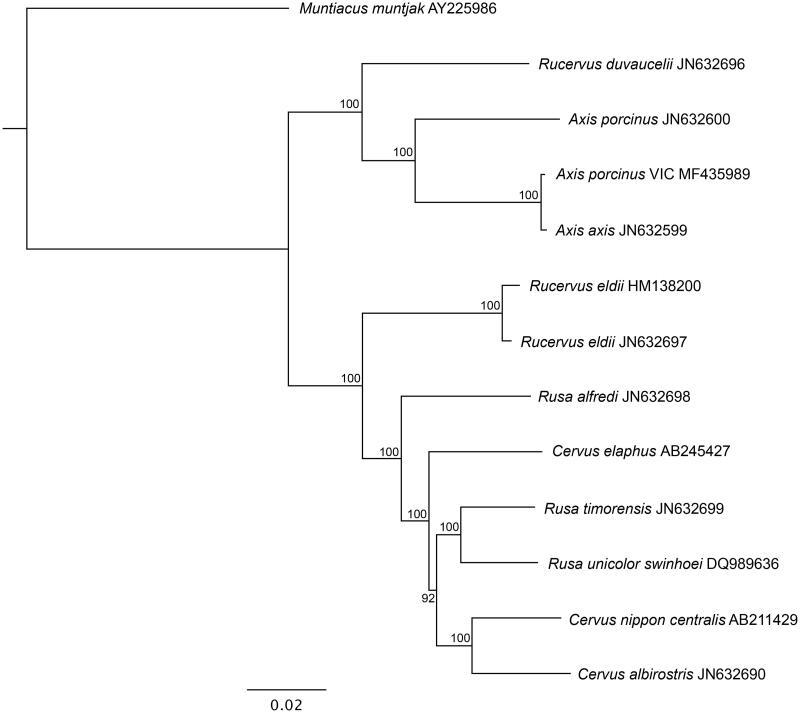
Maximum likelihood phylogenetic tree of complete mitochondrial genomes of Victorian *A. porcinus* and closely related species. GenBank accession numbers are listed next to species’ names. Bootstrap values are listed next to nodes, with 1000 replicates. *Muntiacus muntjak* was used as an outgroup.

The Victorian *A. porcinus* shared a 99.8% similarity to the mitochondrial genome of *A. axis*, and only 94.66% similarity to another *A. porcinus* genome. In comparison, two mitochondrial genomes of *Rucervus eldii* taken from GenBank (HM138200 and JN632697) shared a 99.4% similarity.

No samples of chital were analysed in the lab prior to this experiment, so contamination of samples can be ruled out. Further research is ongoing to understand why the mitochondrial genomes of Victorian *A. porcinus* and *A. axis* are so similar.
